# Truthful Incentive Mechanisms for Social Cost Minimization in Mobile Crowdsourcing Systems

**DOI:** 10.3390/s16040481

**Published:** 2016-04-06

**Authors:** Zhuojun Duan, Mingyuan Yan, Zhipeng Cai, Xiaoming Wang, Meng Han, Yingshu Li

**Affiliations:** 1 Department of Computer Science, Georgia State University, Atlanta, GA 30302, USA; zduan2@student.gsu.edu (Z.D.); mhan7@student.gsu.edu (M.H.); yili@gsu.edu (Y.L.); 2 Department of Computer Science and Information Systems, University of North Georgia, Dahlonega, GA 30597, USA; mingyuan.yan@ung.edu; 3 School of Computer Science, Shaanxi Normal University, Xi’an 710119, China; wangxm@snnu.edu.cn

**Keywords:** Mobile Crowdsourcing Systems (MCSs), incentive mechanism, cost minimization, auction

## Abstract

With the emergence of new technologies, mobile devices are capable of undertaking computational and sensing tasks. A large number of users with these mobile devices promote the formation of the Mobile Crowdsourcing Systems (MCSs). Within a MCS, each mobile device can contribute to the crowdsourcing platform and get rewards from it. In order to achieve better performance, it is important to design a mechanism that can attract enough participants with mobile devices and then allocate the tasks among participants efficiently. In this paper, we are interested in the investigation of tasks allocation and price determination in MCSs. Two truthful auction mechanisms are proposed for different working patterns. A Vickrey–Clarke–Groves (VCG)-based auction mechanism is proposed to the continuous working pattern, and a suboptimal auction mechanism is introduced for the discontinuous working pattern. Further analysis shows that the proposed mechanisms have the properties of individual rationality and computational efficiencies. Experimental results suggest that both mechanisms guarantee all the mobile users bidding with their truthful values and the optimal maximal social cost can be achieved in the VCG-based auction mechanism.

## 1. Introduction

Nowadays, new emerging embedded technology drives the rapid growth of mobile devices. With powerful processors, mobile devices such as smartphones, tablets, and watches can be used as portable computers to undertake heavy computational tasks. With the help of embedded sensors like Global Position System (GPS), accelerometers, and cameras, mobile devices can be used to sense and deliver information. On the other hand, the utilization of mobile devices is ubiquitous. Some works [1,2] show that almost 64% of adults own a smartphone and 42% of adults own a tablet in America as of October 2014. All of the above conditions, along with the mobility of users who carry these mobile devices and the convenient communication infrastructures enable mobile devices to connect to the Internet, stimulating the development of Mobile Crowdsourcing Systems (MCSs) [3]. The MCS is a new system model used to outsource tasks. Generally speaking, two types of participants exist in a MCS. One is the crowdsourcing platform, which acts as the server to publish tasks, determine the set of mobile devices to work on the tasks, and collect the final results. The other participants consist of users with mobile devices. They can participate to finish the tasks published by the crowdsourcing platform and get payments as rewards. Lots of tasks can be done by a MCS, such as information collection, environmental monitoring, or customized survey. These tasks used to be performed by a specialist or an expert, but now through the MCS can be done by a group of undefined users with mobile devices.

A variety of MCS applications can be found in our daily life, among which applications focusing on the environment, infrastructure, and social activities are the three most popular categories. In the environmental MCS applications, such as Common Sense and Creek watch (introduced in [4,5], respectively), mobile devices can be used to monitor the environmental pollution levels. For example, microphones on mobile devices can monitor the noise information of a place and pictures can be taken by cameras to show the amount of trash in a park in Common Sense. Existing applications of the Infrastructure interested in the detection of traffic congestion, parking availability, and outages of public works. For example, mobile devices with CarTel [6] installed on cars can detect the speed and location of cars, and send the detected information to a data center. ParkNet [7] can help cars to find available parking places. Applications regarding social activities take advantage of users’ willingness to share sensed information with each other [8,9]. All MCS applications require the participation of hundreds or thousands of mobile devices without deploying any static sensors or machines.

The enormous utilization potential of MCSs attracts lots of attention from researchers [10,11]. One of the most popular topics in MCSs is how to determine the best set of mobile devices to allocate the tasks (such as computational or sensing tasks) published by a MCS platform so that a predefined objective can be achieved. A commonly used objective is to optimize the social efficiency, such as maximizing social welfare or minimizing social cost. The fundamental of a MCS is to have enough participants. However, working for MCS platforms will consume users’ resources, including execution capacity and battery. Joining a MCS will also put a threat on users’ privacy. For example, the results submitted to MCS platform may expose users’ locations. Considering the above-mentioned facts, some users may refuse to participate in the MCS. If the number of users with mobile devices is insufficient, the objective is impossible to be achieved. Thus, a MCS platform should provide enough reward for participants for incentive purposes.

In this paper, we focus on the design of an incentive mechanism for a MCS to minimize the social cost. The social cost represents the total cost of mobile devices when all tasks published by the MCS are finished. To achieve the objective in the MCS, we confront several challenges:

- *True cost revelation.* The cost of each mobile device for finishing a task is private. It is difficult to encourage all participants to report their real costs.
- *Minimal cost optimization.* Assume all users report their true costs to the MCS. Since mobile devices may vary in capacities and costs, it is hard to select the optimal set of users.
- *Incentive mechanism.* As discussed, the MCS platform should reward each user who works for it as incentives. Within the budget, the reward should be greater than the cost of the user. Deciding a proper reward for each participant is still challenging.

These challenges lead us to investigate an auction mechanism that concentrates on the trade between the MCS platform and mobile users. This paper begins with the assumption that the MCS platform publishes only one task in one round and the task consists of pieces of sub-tasks. Each user with a mobile device can work for one or more sub-tasks. The auction mechanism in our paper aims to minimize the social cost of mobile users while guaranteeing the truthful cost of bidding from each participating user.

Depending on the requirements of a MCS platform, there are two different working patterns. The first one is the *continuous* working pattern, which requires each participant to work on a set of continuous sub-tasks. We call another working pattern the *discontinuous* working pattern, where a participant can work for any set of sub-tasks. The detailed definition of the two working patterns are discussed in Section 4.

The main contributions of this paper are as follows:

1. The social cost minimization problem in a MCS has been discussed. We first present the working process of a MCS, and then build an auction market for the MCS where the MCS platform acts as an auctioneer and users with mobile devices act as bidders.
2. Depending on the different requirements of the MCS platform, we design a Vickrey-Clarke-Groves (VCG)-based auction mechanism for the continuous working pattern and a suboptimal auction mechanism for the discontinuous working pattern. Both of them can ensure that the bidding of users are processed in a truthful way and the utilities of users are maximized.
3. Experiments are conducted to verify performances of the proposed mechanisms. Results suggest that the two auction mechanisms achieve truthfulness and utility maximization. In addition, the VCG-based mechanism could guarantee the minimum social cost and the suboptimal mechanism is more computationally efficient.

The remainder of this paper is organized as follows: related works are shown in Section 2. In Section 3, we present the MCS model and analyze its working process. The auction problem is defined in Section 4, followed by two truthful auction mechanisms presented in Section 5. The experimental results and discussions are provided in Section 6. Conclusions and future works are shown in the last section.

## 2. Related Works

Recently, many commercial MCS applications have been released. These applications can be installed on mobile devices carried by users. After installation, these mobile users are able to undertake computational or sensing tasks. Then, all the results or information generated by the applications will be transmitted to a service center for final process. For example, GigWalk [12] can assist users in verifying the service quality and product placement. It can also provide reports about the graffiti at bus or train stations to the government. Additionally, GigWalk could work for real estate, consumer research, travel, advertising, and so on. Field Agent [13] is an application used for businesses. It can work for two tasks—audit and research. The audit task mainly focuses on information collection, which allows manufacturers and retailers to attract customers and spread information [14,15,16]. The research task is interested in gathering customers’ feedback on products or services, so the businessmen can have a better insight of the market. In [4], the authors introduce a MCS application named Common Sense, which is used for pollution monitoring. Nericell [17] can be used to determine the average speeds or traffic delays, and DietSense [9] is proposed for health control. These applications suggest that the importance of MCS is growing in practical business fields.

Many studies have been done on task allocation in MCSs. Most of them target the maximization of system efficiency. In [18], the authors design a fair energy-efficient allocation framework and propose two sensing task allocation algorithms: one is an offline allocation algorithm and the other is an online allocation algorithm. Ho and Vaughan [19] formalize the online task assignment problem, which makes the allocation decision upon arrival of each worker. Then, a two-phase exploration–exploitation assignment algorithm is proposed. Authors of [20] investigate the problem of task assignment and label inference for heterogeneous classification tasks. They derive a probably near-optimal adaptive assignment algorithm by applying online primal-dual techniques. An architectural model using the SLURM tool for efficient management in the MCS is outlined in [21]. The authors propose a novel idea of adaptive task scheduling which is based on the feedback of customer satisfaction. However, they don’t consider the incentive mechanisms.

A handful of researchers put effort on the design of incentive mechanisms for the MCS. Yang *et al.* [22] consider two types of incentive mechanisms: platform-centric incentive mechanisms and user-centric incentive mechanisms. The first one is based on the Stackelberg game, in which the MCS platform has absolute control over the total budget to users, and users can only adjust their actions to meet the requirements of the platform. The roles of the platform and users are reversed in the user-centric incentive mechanisms. Each user reports the lowest price for selling a service to the MCS platform. In [23], the authors design a reward-based collaboration mechanism. The client publishes a total reward to be shared among collaborators. The collaboration is successful when enough users are willing to collaborate. In order to attract more users to participate, [24] designs a novel Reverse Auction-based Dynamic Price (RADP) incentive mechanism. In this mechanism, users can sell their sensing data to a service provider by their claimed bid prices. Singla and Krause [25] exploit a link between procurement auctions and multi-armed bandits. Its mechanism design is budget feasible. In conclusion, most of the existing works concentrate on maximizing social efficiency and achieving fairness in MCSs.

## 3. System Model Overview

**Figure 1:**
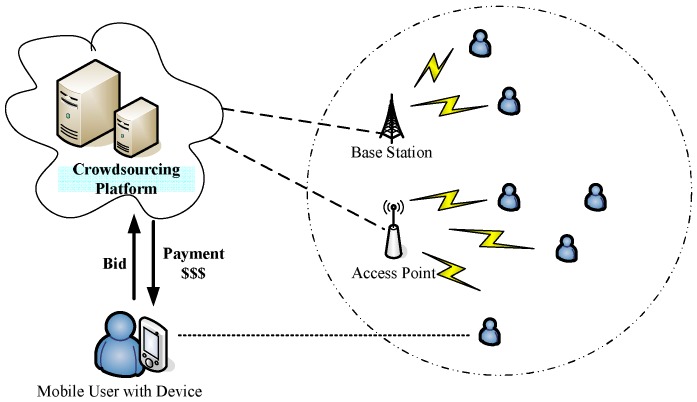
An overview of a Mobile Crowdsourcing System (MCS).

Figure 1 demonstrates an example of the Mobile Crowdsourcing System (MCS). The model includes two types of participants: a Crowdsourcing Platform (CP) and lots of Mobile Users with Devices (MUDs). The CP consists of several servers, which are deployed in the cloud and provide services for clients. A smartphone or a tablet carried by a user is regarded as a MUD. The CP communicates with MUDs via cellular networks or WiFi. The CP publishes a computational or a sensing task which contains a series of sub-tasks. Each sub-task only occupies a time slot. Any two time slots have no intersection. Each MUD is allowed to work on one or more time slots and provide computational or sensing services to the CP during these time slots. The concepts sub-task and time slot are used interchangeably in this paper. Working for computational or sensing tasks will bring battery consumption, computing capacity consumption, and privacy threats to MUDs. Thus, in order to stimulate MUDs’ participation, the CP rewards these users who have been selected to provide serves. For simplicity, we assume that the CP publishes one task in each round.

**Figure 2:**
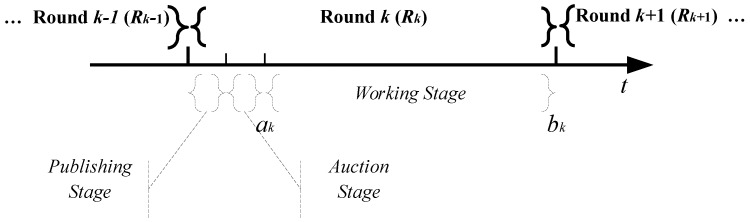
The interactive process between the crowdsourcing platform (CP) and mobile users with devices (MUDs).

In general, as shown in Figure 2, the interactive process between the CP and MUDs have three stages in each round, including the publishing stage, auction stage, and working stage:

*Publishing stage.* In this stage, the CP decides the task that it plans to finish in this round. It generates the description of the task according to predefined functions and then publishes it among all participated MUDs.

*Auction stage.* After receiving the task description and requirements, each MUD decides its working plan. That is the subset of sub-tasks of task *k* it can work for. If a MUD can work for task *k*, it will continue to evaluate its cost. The MUD calculates its base price and submits a bid to the CP. The bid of a MUD consists of its working plan and the base price. After receiving the bids from MUDs, the CP will choose the winner set of MUDs, make the work schedule, determine their rewards and then announce the auction results to all participated MUDs.

*Working stage.* For each task *k*, its working stage starts at the start time $a_{k}$ and ends at the end time $d_{k}$. During this stage, the CP will activate the MUDs in the winner set one by one based on its working schedule. Once activated, a MUD begins to work according to the requirements and then submits the result to the CP. The reward is given to the MUD once it finishes its claimed sub-tasks.

Different from the auction stage, the other three stages in the MCS are beyond the scope of this paper. We focus on designing efficient and effective auction mechanisms. Table 1 lists frequently used notations.

## 4. Problem Formulation in Auction Stage

**Figure 3:**
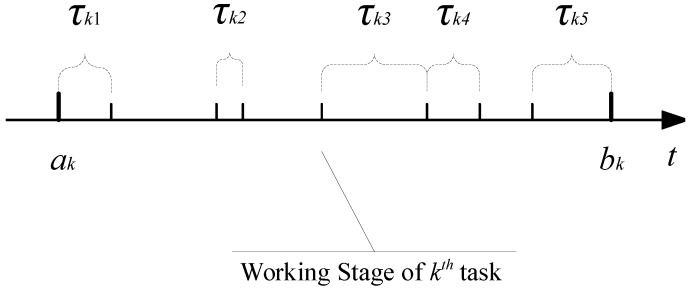
An example of sub-tasks in one round.

The working process of a MCS can be divided into infinite rounds with time. For any two rounds, their tasks are independent and the available MUDs are independent as well. Thus, we focus our investigation on the discussion of one round in detail. Assume there is a dense set of MUDs, represented as $V_{k}=\left\{v_{1},\;v_{2},\;\ldots \;,v_{i},\;\ldots \;,\;v_{N}\right\}$, where $|V_{k}|=N$. At the beginning of round *k* (*k* is an integer denoting the identifier of one round), the CP publishes the task description $R_{k}$, defined as:

$$R_{k}=\left\{a_{k},d_{k},T_{k},\Omega_{k},\;\Pi_{k}\right\},$$

where $a_{k}$ and $d_{k}$ are the start and end time of task *k*, respectively. $T_{k}=\left\{\tau_{k1},\;\tau_{k2},\;\ldots ,\;\tau_{kj},\;\ldots ,\;\tau_{kM}\right\}$ represents the set of sub-tasks of task *k*. Each time slot represents a sub-task of task *k*. *M* is the size of $T_{k}$ (the number of sub-tasks in task *k*). The CP requires MUDs to work for task $T_{k}$. As shown in Figure 3, on the one hand, the durations of any two time slots $\tau_{ki}\in T_{k}$ and $\tau_{kj}\in T_{k}$, where i≠j, may vary from each other. On the other hand, all these time slots are distributed over the time line. The interval between two adjacent time slots could be larger than or equal to 0, but not smaller than 0, which means that no overlapping time interval exists between any two adjacent time slots. $\Omega_{k}$ indicates the hardware requirements of task *k* on MUDs. Hardware requirements contain minimum computation speed, free storage capacities, and sensor types. It requires that only the MUDs satisfying the requirements can bid for the sub-tasks at this round. $\Pi_{k}$ indicates the requirement of task *k* regarding MUDs’ working patterns. There are two kinds of working patterns: the continuous pattern ($\Pi_{k}=C$) and the discontinuous pattern ($\Pi_{k}=\bar{C}$).

*Continuous case (C)*: $\forall \;v_{i}\in V_{k}$, this working pattern requires the sub-tasks set $S_{i}$ claimed by $v_{i}$ are continuous ($S_{i}$ represents the set of sub-tasks $v_{i}$ can work for). That is, $v_{i}$ is able to work continuously from the earliest sub-task to the last sub-task in $S_{i}$. For example, suppose five sub-tasks are included in task *k*, as shown in Figure 3. $S_{i}=\left\{\tau_{k2},\;\tau_{k3},\;\tau_{k4}\right\}$ is an example that $v_{i}$ works in a continuous working pattern, while $S_{i}=\left\{\tau_{k2},\;\tau_{k3},\;\tau_{k5}\right\}$ is not.

*Discontinuous case ($\bar{C}$)*: $\forall \;v_{i}\in V_{k}$, in this case, $v_{i}$ can work for any subset of $T_{k}$. For example, both $S_{i}=\left\{\tau_{k2},\;\tau_{k3},\;\tau_{k4}\right\}$ and $S_{i}=\left\{\tau_{k2},\;\tau_{k3},\;\tau_{k5}\right\}$ can be regarded as examples of discontinuous working patterns.

$\forall \;v_{i}\in V_{k}$, after receiving a task description from the CP, if its hardware qualifies, $v_{i}$ will decide the subset of sub-tasks, denoted as $S_{i}\subseteq T_{k}$ according to the working patterns requirements of task *k*. Then, $\forall \;v_{i}\in V_{k}$, the bid of $v_{i}$ can be represented as,

$$b_{i}=\left\{\Omega_{i},\;\Pi_{i},\;S_{i},\;A_{i}\;\right\},$$

where $A_{i}$ is $v_{i}$’s asking price when it works on the sub-tasks in $S_{i}$ for the CP, which is also the base price. Because auction mechanisms are expected to be truthful, so $A_{i}=c_{i}$. In practice, the value of $c_{i}$ can be estimated by $v_{i}$. $A_{i}$ and $c_{i}$ are used interchangeably in this paper. For convenience, all MUDs’ costs are restricted to follow simple cost functions which makes all MUDs *single-minded MUDs*.

**Definition 1.**  *A cost function C is called* single-minded if there exists a set of sub-tasks $S\subseteq T_{k}$ and a cost c, $C(S^{*})=c$ for all allocations $S^{*}\subseteq S$ and $C(S^{*})=\infty$ for all other $S^{*}$. A MUD bids with S and c is single-minded.

Definition 1 shows that the base price of each $v_{i}$ will be same even though the set of sub-tasks $S_{i}^{*}$ allocated to $v_{i}$ is a subset of $S_{i}$ in its bid after the auction stage. One step further, the MUD $v_{i}$ wouldn’t accept any allocation $S^{*}$, where $\exists \;\tau \in T_{k},\;\tau \in S^{*}\;but\;\tau \notin S$.

In the auction stage, the CP can be regarded as an auctioneer who makes the decision for the sub-tasks allocation and payment. MUDs act as bidders to make bids. We are interested in minimizing the social cost from a macroscopic and social perspective in this paper. The social cost is defined as the cost brought by the trading within the MCS. Formally, the objective can be written as,

(1) $$\begin{array}{c} Minimize\;\;\;\sum_{v_{i}\in W_{k}}\left(P_{i}+\left(c_{i}-p_{i}\right)\right)\\ \;\;\;\;\;\;s.t.\;\;\;W_{k}\subseteq V_{k},\;\;T_{k}=\underset{v_{i}\in W_{k}}{⋃}S_{i} \end{array}$$

where $W_{k}$ is the set of winner MUDs in this round. $P_{i}$ is the price paid by the CP for using the computational or sensing services provided by $v_{i}$, which can be regarded as the cost of the CP. $p_{i}$ is the payment winner $v_{i}$ received from the CP when the assigned sub-tasks are done, and $c_{i}$ is the cost of $v_{i}$. So, the social cost of winner $v_{i}$ is $c_{i}-p_{i}$. For effectiveness, each sub-task *τ* in $T_{k}$ needs at least one MUD to work.

Based on the predefined model, we have $P_{i}=p_{i}$ for each $v_{i}\in W_{k}$. Hence, the objective above can be rewritten as

(2) $$\begin{array}{c} Minimize\;\;\;\;\;\sum_{v_{i}\in W_{k}}c_{i},\\ \;\;\;\;\;\;\;\;s.t.\;\;\;W_{k}\subseteq V_{k},\;\;T_{k}=\underset{v_{i}\in W_{k}}{\cup }S_{i}. \end{array}$$

Within the auction stage, the auctioneer CP should design a proper auction mechanism with efficient sub-tasks allocation and rewards determination to minimize the social cost. Under the auction mechanism chosen by the CP, each participated MUD bids with a strategy which maximizes its utility. The utility of $v_{i}$ (denoted by $U_{i}$) in one round can be defined as:

(3) $$U_{i}=\left\{\begin{array}{c} p_{i}-c_{i}\;\;\;\;v_{i}\;wins,\\ 0\;\;\;\;otherwise. \end{array}\right.$$

In order to achieve the objective in an efficient and effective way, the auction mechanisms used by the CP should have the following properties:

**Individual Rationality.** All MUDs are self-interested to benefit themselves. Thus, the utility of any MUD in each round should be non-negative: $U_{i}\geq 0$.

**Truthfulness.** The mechanisms are considered truthful when the four values ($\Omega_{i},\;\Pi_{i},\;S_{i},\;A_{i}$) in the bid of each MUD are truthful. The utility of $v_{i}$ will be maximized when it bids truthfully and $v_{i}$ cannot improve its utility through any misreport,

(4) $$U_{i}\left(b_{i},b_{-i}\right)\geq U_{i}\left({\hat{b}}_{i},b_{-i}\right),$$

where $b_{-i}=\left\{b_{1},\;\ldots \;,\;b_{i-1},\;b_{i+1},\;\ldots \;,\;b_{n}\;\right\}$ represents the set of truthful bids of all MUDs excluding $v_{i}$. $b_{i}$ is the truthful bid of $v_{i}$, and ${\hat{b}}_{i}\neq b_{i}$. If an auction mechanism satisfies this property, NashEquilibrium exists [26]. Misreports of the first two values (hardware parameters and working pattern of a MUD) in a bid can be easily detected by the CP through the submitted results from MUDs. Thus, the truthfulness of the first two values is guaranteed. We focus on the truthfulness of the last two values in a bid: claimed set of sub-tasks and asking price.

**Computational Efficiency.** An auction mechanism is considered computationally efficient if the task allocation and payment decision can be made in polynomial time.

Only when the above three properties are satisfied at the same time can an auction mechanism be regarded as useful. Without individual rationality, a MUD may receive negative utility and refuse to participate in the MCS. Then, because the $c_{i}$ in bid $b_{i}$ is private to $v_{i}$, the CP wouldn’t know it. If an auction mechanism is truthful, all MUDs only need to bid with their true costs: $A_{i}=c_{i}$, which not only simplifies the strategies, but also avoids manipulation. Finally, computational efficiency will guarantee that the auction mechanism can be practically implemented.

## 5. Design of Incentive Auction Mechanisms

Formally, an auction mechanism contains two phases: winner MUDs set the selection and payment decision. Specifically, the most challenging and important part of the auction mechanism design is truthfulness. According to the characterization of truthful auction mechanism concluded in [26], we have:

**Theorem 2.**  For any fixed bids $b_{-i}$, an auction mechanism is truthful to MUD $v_{i}$ if and only if the winner MUDs set selection algorithm is monotone and the payment for each winner MUD is critical.

**Definition 3.**  If the MUD $v_{i}$ is selected as a winner when it bids with $S_{i}^{*}$ and $A_{i}^{*}$, and $v_{i}$ will still be selected for any ($S_{i}^{\prime }$, $A_{i}^{\prime }$), where $S_{i}^{\prime }⊇S_{i}^{*}$ and any bid with $A_{i}^{\prime }\leq A_{i}^{*}$. The process in the selection of winner MUDs set is monotone.

**Definition 4.**  Critical payment. There exists a critical payment ${c^{c}}_{i}$ for each winner MUD $v_{i}$, which is independent to the asking price $a_{i}$ in its bid. $v_{i}$ will win when it bids with any ($S_{i}$, $A_{i}^{\prime }$), where $A_{i}^{\prime }\leq {c^{c}}_{i}$. Otherwise, $v_{i}$ loses.

### 5.1. Mechanism with Optimal Social Cost

Based on Section 4, we can tell that different tasks may have different working pattern requirements. If a task *k* requires MUDs to work in the continuous pattern ($\Pi_{k}=C$), the optimal solution to the minimization Problem (2) can be achieved through dynamic programming. Thus, in this case, a well known VCG-based auction mechanism is a good choice. The detailed design is as follows:

*Step 1,* Given the bids of all candidate MUDs, use the dynamic programming method, shown in Algorithm 1, to compute the optimal winner set *W*. In Algorithm 1, Line (8) represents the optimal substructure, recording the minimal cost for sub-task *τ* in *T* if adding the $v_{n}$ to the winner set *W*. Notation τ.MUD is used to indicate that the winner MUD will work for the sub-task *τ*. This algorithm returns three results: the winner set *W*, the work schedule, and minimal social cost of MUDs in *W*.

The complexity of Algorithm 1 is O(mn), which indicates that the VCG-based auction is practical.

*Step 2,* Calculate the payment $p_{i}$ of each $v_{i}$ in the winner set *W*. The payment for each winner $v_{i}$ is defined as the increase in the total social cost brought by its contribution, as

(5) $$p_{i}=\sum_{v_{j}\in W_{-i}}c_{j}\;-\sum_{v_{j}\in W,\;v_{j}\neq v_{i}}c_{j},$$

where $W_{i}$ is the obtained winner set without $v_{i}$’s participation.

The truthfulness and individual rationality of VCG-based mechanisms have been proven in [26].

### 5.2. Mechanism with Suboptimal Social Cost

If the working pattern is discontinuous, the winner set determination Problem (2) can be regarded as a classical set cover problem. Because the set cover problem has been proven to be NP-hard, it is impossible to obtain the optimal solution in a MCS with large scale. Without optimal winner selection, truthfulness cannot be guaranteed by VCG-based auction mechanism. Therefore, we propose another mechanism with suboptimal social cost, acceptable computational complexity, and truthfulness.

The detailed winner set determination algorithm is shown in Algorithm 2. It consists of two steps. First, sort all MUDs in ascending order, according to their average cost $\frac{c_{i}}{|S_{i}|}$ (as shown in line (2)). Then, MUDs will be added to winner set *W* one by one, according to the ascending order derived in the last step, until all sub-tasks in *T* are covered, as shown in lines (4–13). The payment of each winner $v_{i}$ is determined based on Algorithm 3. Specifically, it first reorders all MUDs, excluding $v_{i}$, in ascending order based on their average cost $\frac{c_{j}}{|S_{j}|}$ (as shown in line (2)). Then find the least position *j* in the order that: $v_{i}$ may lose in the case of $\frac{c_{i}}{|S_{i}|}>\frac{c_{j}}{|S_{j}|}$.

**Algorithm 1** Optimal Winner MUDs Set Selection**Input:**    Set of sub-tasks *T*, |T|=M.    ($S_{1}$, $c_{1}$), ..., ($S_{i}$, $c_{i}$), ..., ($S_{N}$, $c_{N}$),    *Working pattern: continuous.***Output:**    miniCost, Winner set *W*;    Working schedule τ.MUD,∀τ∈T.1:*Initialization:*2:**for** each *τ* in *T*
**do**3:  $\tau_{cost}$= MAX.VALUE;4:**end for**5:**for** each *τ* in *T*
**do**6:  **for** each $v_{i}$ in *V*
**do**7:      **if**
*τ* is in $S_{i}$
**then**8:            $currentMini=(\tau_{0}==\tau_{i}=S_{i}.first$)? $c_{i}:\left(c_{i}+\tau_{i-1}.cost\right)$;9:      **end if**10:      **if**
currentMini<τ.cost
**then**11:            τ.cost=currentMini;12:            $\tau .MUD=v_{i}$;13:            **for** each $\tau^{\prime }$ in $S_{n}$
**do**14:                    $\tau^{\prime }.cost=currentMini$;15:                    $\tau^{\prime }.MUD=v_{i}$;16:            **end for**17:      **end if**18:  **end for**19:**end for**20:**for** each *τ* in *T*
**do**21:  Put τ.MUD into *W*;22:**end for**23:miniCost=T.last.cost;

**Lemma 5.**  *The winner MUDs set selection provided in Algorithm 2 is monotonic.*

**Proof.**  Suppose $v_{i}$ is selected as a winner by bidding with $c_{i}$ and $S_{i}$, its average cost is $\alpha_{i}=\frac{c_{i}}{|S_{i}|}$. Let $c_{i}^{\prime }\leq c_{i}$, and $S_{i}$ remain unchanged, we should prove that $v_{i}$ would still win when bidding with $c_{i}^{\prime }$ and $S_{i}$. The new average cost is $\alpha_{i}^{\prime }=\frac{c_{i}^{\prime }}{|S_{i}|}$. Since $\alpha_{i}^{\prime }\leq \alpha_{i}$, $v_{i}$ will be selected earlier by Algorithm 2. It is easy to know that $v_{i}$ will continue to win with bidding with $c_{i}$ and $S_{i}^{\prime }$, where $S_{i}^{\prime }⊇S_{i}$. For the reason that the new average cost $\alpha_{i}^{\prime }=\frac{c_{i}}{|S_{i}^{\prime }|}$ is smaller than $\alpha_{i}=\frac{c_{i}}{|S_{i}|}$. ☐

**Lemma 6.**  The payment $p_{i}$ to each $v_{i}\in W$ is equal to its critical cost $c_{i}^{c}$.

**Proof.**  Assume that critical cost of each MUD is equal to its payment, that is, $\forall \;v_{i}\in W,\;c_{i}^{c}=p_{i}$. Based on Algorithm 3, $c_{i}^{c}=p_{i}=\frac{c_{j}}{|S_{j}|}\left|S_{i}\right|$, where $S_{i}\subset \underset{i^{\prime }=1,\ldots ,j}{⋃}S_{i^{\prime }}$. If $v_{i}$ bids with $c_{i}^{\prime }>c_{i}^{c}$, then $\frac{c_{i}^{\prime }}{\left|S_{i}\right|}>\frac{c_{j}}{|S_{j}|}$, indicating that the average cost of $v_{i}$ is larger than the average cost of $v_{j}$, the position of $v_{j}$ in this new order is before $v_{i}$. In this case, once the $v_{j}$ is in *W*, $v_{i}$ will have no chance to be selected as a winner. On the other hand, if $v_{i}$ bids with $c_{i}^{\prime }\leq c_{i}^{c}$, it will still be a winner according to Lemma 5. Thus the assumption is verified. ☐

**Theorem 7.**  The suboptimal cost mechanism is truthful.

**Algorithm 2** Suboptimal Winner Set Selection**Input:**    Set of sub-tasks *T*.    ($S_{1}$, $c_{1}$), ..., ($S_{i}$, $c_{i}$), ..., ($S_{N}$, $c_{N}$),**Output:**    MiniCost, Winner set *W*;    Working schedule τ.MUD,∀τ∈T.1:*Initialization:*2:$\forall \;v_{i}\in V$, sort ascendingly:
$$\frac{c_{1}}{|S_{1}|}\leq \frac{c_{2}}{|S_{2}|}\leq \ldots \leq \frac{c_{N}}{|S_{N}|},$$3:U=∅, j=1;4:**while**
U≠T
**do**5:  **if**
$U\cap S_{j}!=S_{j}$
**then**6:      put $v_{j}$ into *W*, $U=U⋃S_{j}$,7:      $miniCost=miniCost+c_{j}$;8:  **end if**9:  **for** Each *τ* in $S_{j}$
**do**10:      $\tau .MUD=v_{j}$;11:  **end for**12:  j++;13:**end**
**while**

**Algorithm 3** Price Determination**Input:**    Set of sub-tasks *T*,    ($S_{1}$, $c_{1}$), ..., ($S_{i}$, $c_{i}$), ..., ($S_{N}$, $c_{N}$),    Winner set *W*;**Output:**    Price $p_{i},\forall v_{i}\in W$;1:**for** each $v_{i}$ in *W*
**do**2:  $\forall \;v_{j}\in V\backslash v_{i}$, sort ascendingly:
$$\frac{c_{1}}{|S_{1}|}\leq \frac{c_{2}}{|S_{2}|}\leq \ldots \leq \frac{c_{N-1}}{|S_{N-1}|};$$3:  Find the least index *j* that $S_{i}\subset \underset{i^{\prime }=1,\ldots ,j}{⋃}S_{i^{\prime }}$;4:  The payment for MUD $v_{i}$ is:
$$p_{i}=\frac{c_{j}}{|S_{j}|}\left|S_{i}\right|;$$5:**end**
**for**

**Proof.**  With Lemmas 5 and 6, this theorem can be proven based on Theorem 2. ☐

**Theorem 8.**  *The suboptimal cost mechanism is individual rational.*

**Proof.**  For $\forall \;v_{i}\in V_{k}$, its payment $p_{i}$ is equal to its critical cost $c_{i}^{c}$. If $v_{i}$ wins, there must be $c_{i}\leq c_{i}^{c}$. Hence, $U_{i}=p_{i}-c_{i}\geq 0$. Or $v_{i}$ loses, its utility is 0. Individual rationality is guaranteed. ☐

**Theorem 9.**  *The suboptimal cost mechanism is computationally efficient.*

**Proof.**  The complexity of Algorithm 2 is $O(n^{2})$. The complexity of Algorithm 3 is $O(n^{2})$. The suboptimal social cost auction mechanism can be implemented with an acceptable time complexity. ☐

## 6. Performance Evaluation

To evaluate the performance of the incentive auction mechanisms proposed in this paper, experiments are conducted.

### 6.1. Continuous Working Pattern

We set the number of sub-tasks in one round to vary from 50 to 100. The number of MUDs is fixed as 30 (*F* = 30). Each selects a subset of continuous sub-tasks and the size of the subset is randomly chosen from [3, 15]. The cost of each MUD is distributed over [10, 15] uniformly. The VCG-based auction mechanism is designed for the continuous working pattern. Because the continuous case can be regarded as a special case of discontinuous working pattern, the suboptimal auction mechanism can also be used to solve the problem here. Both the VCG-based auction mechanism and suboptimal auction mechanism are evaluated in this section.

**Figure 4:**
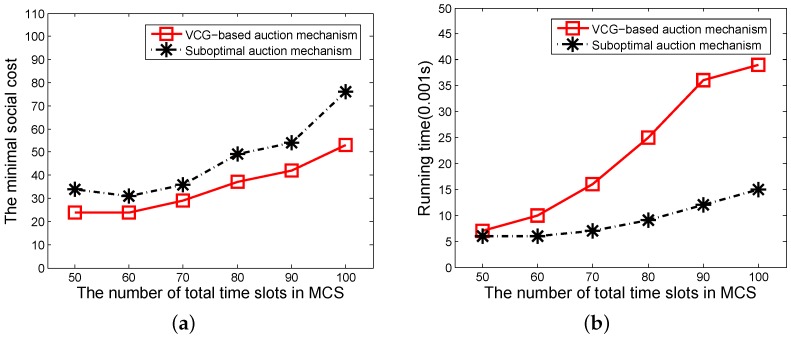
(**a**) The social cost of two auction mechanisms in continuous working pattern; (**b**) The running time of two auction mechanisms in continuous working pattern. VCG: Vickrey–Clarke–Groves.

In this experiment, the number of sub-tasks changes from 50 to 100, the remaining parameters are set as above. We first compare the performances of the VCG-based auction mechanism and the suboptimal auction mechanism regarding the social cost and running time. The results of the social costs of the two auction mechanisms are shown in Figure 4a. The social cost of the VCG-based mechanism is smaller than the suboptimal auction mechanism because the dynamic winner set selection algorithm used in the VCG-based mechanism can find the subset of MUDs which is able to minimize the social cost. However, when considering the running times, as shown in Figure 4b, the suboptimal auction mechanism outperforms the VCG-based auction mechanism. We can also observe that with the increase of the number of total sub-tasks, both the cost and running time increase. When the number of sub-tasks increases, the running time of the VCG-based auction mechanism and the suboptimal auction mechanism increase by 700% and 200%, respectively. However, the difference of social costs between two mechanisms keeps steady at the same time. Thus, it is better to use the VCG-based auction mechanism in the continuous working pattern on the condition that a task has fewer sub-tasks.

**Figure 5:**
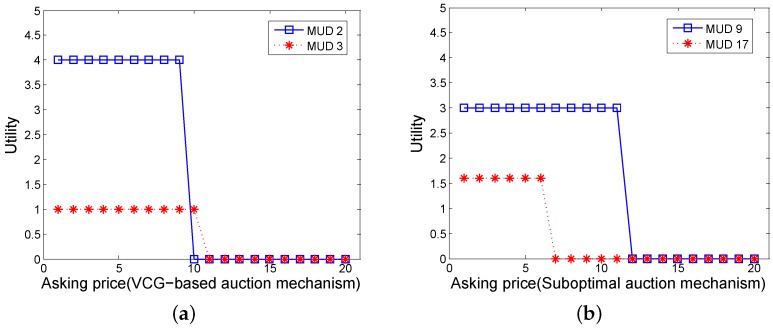
(**a**) The utility of MUD 2 and MUD 3 by VCG-based auction mechanism in continuous working case; (**b**) The utility of MUD 9 and MUD 17 by suboptimal auction mechanism in continuous working case.

Then we try to observe the utilities of winner MUDs by the VCG-based auction mechanism. For simplicity, we choose two MUDs randomly, denoted as MUD 2 and MUD 3. We allow the two MUDs to ask different prices and show the truthfulness in Figure 5a. It is shown that both the MUD 2 and MUD 3 will reach their maximal utilities when they ask the prices truthfully: $A_{2}=c_{2}=10$ and $A_{3}=c_{3}=9$. Figure 5b presents the utilities of MUD 9 and MUD 17 in the suboptimal auction mechanism. Note that MUD 9 and MUD 17 are chosen randomly too. Similarly, they will also achieve their maximal utilities when acting truthfully: $A_{9}=c_{9}=9$ and $A_{17}=c_{17}=5$.

### 6.2. Discontinuous Working Pattern

**Figure 6:**
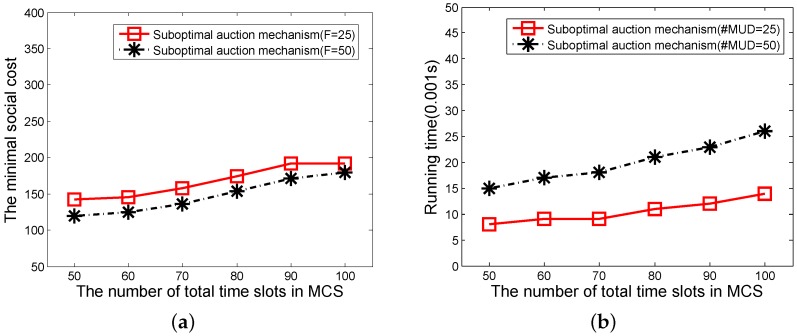
(**a**) The social cost of suboptimal auction mechanism in discontinuous working pattern; (**b**) The running time of suboptimal auction mechanism in discontinuous working pattern.

The experimental setting of the discontinuous working pattern is similar to the continuous working pattern. Besides, each MUD could select the subset of sub-tasks randomly. Figure 6a,b shows the results of suboptimal auction mechanism of the social cost and running time when the number of MUDs is 30 and 50 (*F* = 25 and *F* = 50), respectively. Because the more sub-tasks that are contained within a task, the more works need to be done. We can see that when the number of sub-tasks of a task changes from 50 to 100, both the social cost and the running time increase (see Figure 6a,b). So, it is a trade-off between objective and efficiency in the suboptimal auction mechanism.

**Figure 7:**
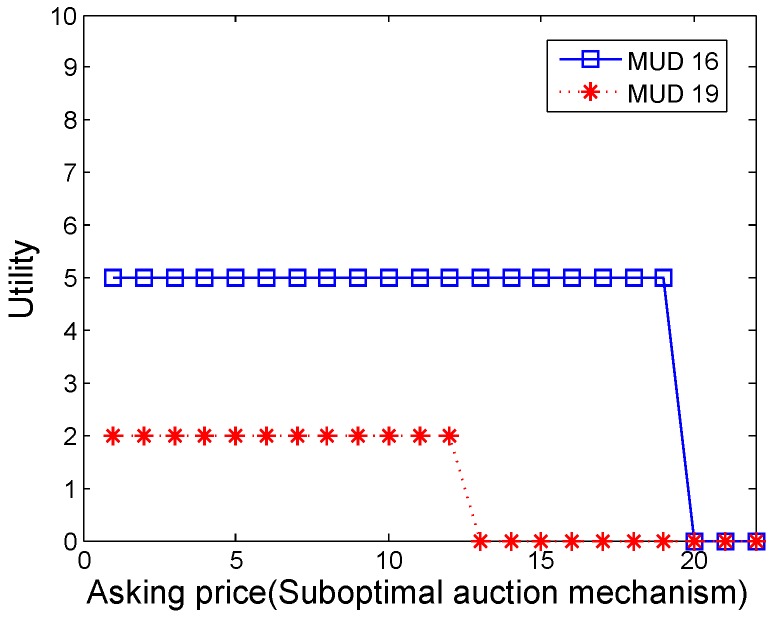
The utility of MUD 16 and MUD 19 by suboptimal auction mechanism in discontinuous case.

Then we try to verify the truthfulness of the suboptimal auction mechanism in the discontinuous working pattern. MUD 16 and MUD 19 are picked randomly, where $c_{16}=14$ and $c_{19}=10$. Let the two MUDs ask different prices from their true costs, their utilities are shown in Figure 7. We can see that both MUDs will get their maximal utilities when asking prices truthfully.

## 7. Conclusions

In this paper, we investigate the incentive auction mechanisms for mobile crowdsourcing systems. We consider two working patterns in works allocation: the continuous working pattern and the discontinuous working pattern. The objective of the MCS platform is to minimize the social cost in both cases. To achieve the truthfulness, individual rationality, and computational efficiency, we design a VCG-based auction mechanism for the continuous case and a suboptimal auction mechanism for the discontinuous case. We have proven that the two mechanisms can implement the three properties simultaneously. In the future, we plan to design an online incentive mechanism to minimize the social cost and try to maximize the utility of each participated MUD.

## Figures and Tables

**Table 1:** Table of Notations.

Notation	Description
CP	Crowdsourcing Platform
MUD	Mobile User Device
*V*, $v_{i}$	set of MUDs and MUD
*k*	round and task identifier
$R_{k}$	description of task *k*
$a_{k}$, $d_{k}$	the start time and end time of task *k*
$T_{k}$,	set of sub-tasks in task *k*
$\tau_{ki}$, *τ*	sub-task in task *k*, sub-task
$\Omega_{k}$, $\Pi_{k}$	hardware parameters and working patterens
$b_{i}$	bid of MUD $v_{i}$
$b_{-i}$	bids of all MUDs except $v_{i}$
$c_{i}$, $p_{i}$, $U_{i}$	cost, payment and utility of MUD $v_{i}$
$A_{i}$	asking price of MUD $v_{i}$
$S_{i}$	subset of sub-tasks MUD $v_{i}$ can work for
$W_{k}$	set of winner MUDs
*F*	number of MUDs

## References

[1] Mobile Technology Fact Sheet. http://www.pewinternet.org/fact-sheets/mobile-technology-fact-sheet/.

[2] Chatzimilioudis G., Andreas K., Christos L., Demetrios Z.-Y. (2012). Crowdsourcing with smartphones. IEEE Internet Comput..

[3] Fuchs-Kittowski F., Daniel F. (2014). Architecture of mobile crowdsourcing systems. Collaboration and Technology.

[4] Dutta P., Aoki P.M., Kumar N., Mainwaring A., Myers C., Willett W., Woodruff A. (2009). Common sense: Participatory urban sensing using a network of handheld air quality monitors. Proceedings of the 7th ACM Conference on Embedded Networked Sensor Systems.

[5] Kim S., Robson C., Zimmerman T., Pierce J., Haber E.M. Creek watch: Pairing usefulness and usability for successful citizen science. Proceedings of the SIGCHI Conference on Human Factors in Computing Systems.

[6] Hull B., Bychkovsky V., Zhang Y., Chen K., Goraczko M., Miu A., Shih E., Balakrishnan H., Madden S. CarTel: A distributed mobile sensor computing system. Proceedings of the 4th International Conference on Embedded Networked Sensor Systems.

[7] Mathur S., Jin T., Kasturirangan N., Chandrasekaran J., Xue W., Gruteser M., Trappe W. Parknet: Drive-by sensing of road-side parking statistics. Proceedings of the 8th International Conference on Mobile Systems, Applications, and Services.

[8] Eisenman S.B., Miluzzo E., Lane N.D., Peterson R.A., Ahn G.S., Campbell A.T. (2009). BikeNet: A mobile sensing system for cyclist experience mapping. ACM Trans. Sens. Netw..

[9] Reddy S., Parker A., Hyman J., Burke J., Estrin D., Hansen M. Image browsing, processing, and clustering for participatory sensing: Lessons from a DietSense prototype. Proceedings of the 4th Workshop on Embedded Networked Sensors.

[10] Li J., Cai Z., Yan M., Li Y. Using crowdsourced data in location-based social networks to explore influence maximization. Proceedings of the 35th Annual IEEE International Conference on Computer Communications, INFOCOM.

[11] Wang Y., Cai Z., Ying G., Dong Y. (2016). An incentive mechanism with privacy protection in mobile crowdsourcing systems. Comput. Netw..

[12] Gigwalk. http://www.gigwalk.com/.

[13] Field Agent. https://fieldagent.net/.

[14] Han M., Yan Y., Cai Z., Li Y. (2016). An exploration of broader influence maximization in timeliness networks with opportunistic selection. J. Netw. Comput. Appl..

[15] Wang X., Lin Y., Zhao Y., Zhang L., Liang Y., Cai Z. (2016). A novel approach for inhibiting misinformation propagation in human mobile opportunistic networks. P2P Netw. Appl..

[16] He Z., Cai Z., Wang X. Modeling propagation dynamics and developing optimized countermeasures for rumor spreading in online social networks. Proceedings of the 2015 IEEE 35th International Conference on Distributed Computing Systems (ICDCS).

[17] Mohan P., Padmanabhan V.N., Ramjee R. Nericell: Rich monitoring of road and traffic conditions using mobile smartphones. Proceedings of the 6th ACM Conference on Embedded Network Sensor Systems.

[18] Zhao Q., Zhu Y., Zhu H., Cao J., Xue G., Li B. Fair energy-efficient sensing task allocation in participatory sensing with smartphones. Proceedings of the 2014 IEEE INFOCOM.

[19] Ho C.J., Vaughan J.W. Online task assignment in crowdsourcing markets. Proceedings of the Twenty-Sixth AAAI Conference on Artificial IntelligenceToronto.

[20] Ho C.J., Jabbari S., Vaughan J.W. Adaptive task assignment for crowdsourced classification. Proceedings of the 30th International Conference on Machine Learning (ICML-13).

[21] Nunia V., Kakadiya B., Hota C., Rajarajan M. (2013). Adaptive task scheduling in service oriented crowd using slurm. Distributed Computing and Internet Technology.

[22] Yang D., Xue G., Fang X., Tang J. Crowdsourcing to smartphones: Incentive mechanism design for mobile phone sensing. Proceedings of the 18th Annual International Conference on Mobile Computing and Networking.

[23] Duan L., Kubo T., Sugiyama K., Huang J., Hasegawa T., Walrand J. Incentive mechanisms for smartphone collaboration in data acquisition and distributed computing. Proceedings of the 2012 IEEE INFOCOM.

[24] Lee J.S., Hoh B. Sell your experiences: A market mechanism based incentive for participatory sensing. Proceedings of the 2010 IEEE International Conference on Pervasive Computing and Communications (PerCom).

[25] Singla A., Krause A. (2013). Truthful incentives in crowdsourcing tasks using regret minimization mechanisms. Proceedings of the 22nd International Conference on World Wide Web.

[26] Vazirani V.V., Roughgarden T., Tardos E. (2007). Algorithmic Game Theory.

